# Perceptions of Safety and Stress Among Health Professionals: The Role of Care Unit Identification as a Protective Factor During the COVID-19 Pandemic

**DOI:** 10.3389/fpsyg.2022.863581

**Published:** 2022-05-30

**Authors:** Chiara Panari, Luca Caricati, Gaetano Gallo, Chiara Bonetti, Alice Bonini, Nadia Monacelli, Alfonso Sollami

**Affiliations:** ^1^University of Parma, Department of Economics and Management, Parma, Italy; ^2^University of Parma, Department of Humanities, Social Sciences and Cultural Industries, Parma, Italy; ^3^Azienda Unità Sanitaria Locale of Piacenza, Piacenza, Italy; ^4^University Hospital Parma Medical Center, Parma, Italy

**Keywords:** health professionals, work engagement, teamwork identification, perception of safety climate, burnout, distress

## Abstract

The present study aimed to investigate the role of motivational process and coping resources in health professionals during the COVID-19 emergency examining the role of Care Unit Identification and safety climate perception as resources that can help nurses to cope with stressors. A cross-sectional research design was used and 218 nurses completed a self-report questionnaire measuring: Perception of safety, Care Unit identification, Work Engagement, Psychological Distress, and Burnout. Results revealed that Work Engagement was significantly related with Burnout (*b* = −0.209, 95%CI [−0.309; −0.109]) and Distress (*b* = −0.355, 95%CI [−0.529; −0.18]) especially when the Care Unit identification is high (*b* = −0.303, 95%CI [−0.448; −0.157] and *b* = −0.523, 95%CI [−0.772; −0.275], respectively). The safety perception was positively related to Work Engagement (*b* = 0.315, 95%CI [0.198; 0.433]) and had an indirect effect on psychological Distress (*b* = −0.112, 95%CI [−0.181; −0.042]) and Burnout (*b* = −0.066, 95%CI [−0.105; −0.027]). High levels of both Care Unit identification and perception of safety, along with personal work engagement, appear to protect nurses from burnout and psychological distress. Findings suggest that the effort to improve teamwork identification and ensures an adequate degree of perceived safety for healthcare professionals need to be maintained and reinforced as they positively impact nurses’ wellbeing.

## Introduction

Since February 2020, health workers involved in the fight against the pandemic have faced a previously unthinkable reality. They have been forced to take complex and difficult decisions, with strong physical, emotional, and psychological pressures. The stressful working conditions resulting from prolonged working hours, the high numbers of serious patients in need of treatment in atypical conditions, the unusual amount of bad news that has had to be communicated to their family members, and the social auto-isolation that was necessary to shield our relatives from possible contagion have strongly influenced the psychological state expressed by health workers (Bertelli et al., 2020). It is crucial that the impact of the pandemic on health professionals, their symptoms of discomfort, risk factors, and coping resources should be recognized and understood.

### Stress Symptoms and Burnout in Health Professionals Resulting From the Pandemic

The nursing category was certainly one of the most affected by the emergence of COVID-19. Although the potential for contagion is present in every living and working environment, healthcare workers are at the greatest risk of exposure to the virus, and their commitment at the forefront of the health emergency also exposes them to increasing operational and emotional overload. In addition to the psychological effects of the state of emergency, health workers have experienced other unique problems and have been exposed to situations of distress with limited possibilities for resolution (Bertelli et al., 2020; Jun et al., 2020). They have had to face psychological stress due to the nature of their job. They have had to deal with a new contagious disease, be in close contact with infectious patients for long durations, redefine the care process with new working procedures or in different environments, and for some, separate themselves from their families to preserve them from possible contagion (Lai et al., 2020).

The pandemic has led to the continuous transformation of strategies, especially in areas with a high COVID-19 prevalence, and this has required workers to adapt accordingly. In addition, the exposure to biological risk, difficulties in finding personal protective equipment (PPE), the excessive workload, irregular work shifts, and anxiety about one’s health have contributed to the development of stress or burnout (Lai et al., 2020).

Stress has been defined in the literature (Lazarus, 1966; Caprara and Borgogni, 1988) as a personal response to external or internal stimulation (stressors) in which the individual tries to restore balance and adapt to the environment. Burnout is defined as a syndrome that occurs more frequently within the caring profession. It is characterized by emotional exhaustion, depersonalization, and reduced professional efficacy (Maslach, 1982). Difficulties in coping with internal or external demands lead to a lack of self-efficacy as the demands of the job exceed the resources the person himself believes he or she possesses. Therefore, while stress typically constitutes a momentary reaction to the need for adaptation, job burnout is chronic (Schaufeli and Enzmann, 1998).

Theoretical approaches emphasize the role of personal, social, and organizational variables in the etiology of burnout and the prevention of possible negatives outcomes. For example, the organizational context defines the constraints and resources available for the worker, the quality of health assistance, the nature and value of relationships with patients and colleagues (Liu et al., 2020), and perceptions of being able to rely on personal abilities and social resources prevent negative health outcomes and generate positives ones (Oshio et al., 2018). The job demands-resources (JDR) model developed by Demerouti et al. (2001) can be used to understand the antecedents of burnout and to predict health workers’ level of wellbeing.

### JDR Model

Demerouti et al. (2001) state that the balance between positive characteristics called resources and negative ones defined as demands can explain the particular job performed by professionals. They describe job demands as “those physical, social, or organizational aspects of work that require physical or mental effort, and are therefore associated with certain physiological and psychological costs” (p. 501). Job resources, on the other hand, are described as “those physical, social, or organizational aspects of work that are characterized by one or more of the following aspects: they are functional to the achievement of job objectives; they reduce job demands and associated physiological and psychological costs; and they stimulate personal growth and development” (p. 501).

Demands and resources are part of the motivational process. Job resources satisfy the individual’s psychological needs, such as autonomy or competence, and determine the extent of their commitment and motivation. Without adequate resources, the individual might be unable to cope with the demands and achieve their goals and even engage in withdrawal behaviors. Resources can therefore play a protective role by mitigating the negative effects of work demands.

It is widely accepted that burdensome job demands (such as excessive workloads or disruptions to the work–life balance) and insufficient job resources (e.g., social support, autonomy, learning opportunities, and feedback) can predict burnout. Conversely, sufficient resources can help the individual deal with the demands of the job and encourage engagement (Schaufeli et al., 2009). High levels of energy and dedication to work have a positive influence on health and performance (Bakker et al., 2008). The JDR model explains how resources and work engagement promote personal growth. It recommends the use of the tools needed to achieve objectives and to cope with job demands, thus reducing the likelihood of stress and burnout (Bakker and Demerouti, 2014). The present study focused on perceptions of safety and teamwork identification.

### Perception of Safety and Care Unit Identification as Protective Resources

The JDR model shows how a secure working environment can be a motivational resource in emergencies. Zohar (1980) defined a climate of safety as “a summary of the molar perceptions that employees share around their work environments” (p.96). During the pandemic, health organizations stressed the importance of protective practices in ensuring the safety of workers and patients; at the same time, staff was asked to work faster (Manzano García and Ayala Calvo, 2020). Nurses were at high risk of exposure to the virus because they were providing intensive health assistance. Perceptions of safety may be framed in terms of individual protection, training and supervision, information sharing, problems with colleagues, and scrupulousness in following contamination prevention procedures (Lai et al., 2020).

The degree of certainty that adequate protection would be provided to nursing staff influenced their capacity to cope (Fernandez et al., 2020). By contrast, uncertainty and disagreements about appropriate infection control measures had negative outcomes (Holroyd and McNaught, 2008). Insufficient investment in safety-related resources was another concern (Ives et al., 2009; Kang et al., 2018).

The pandemic had a strong impact on the quality of the nurses’ working life. They no longer perceived their workplace to be a safe environment, and this threatened their psychological wellbeing (Ahmed et al., 2020).

During emergencies, a sense of belonging to a supportive team becomes very important (Kang et al., 2018). The literature has shown such identification was a predictor of satisfaction among nurses and that a feeling of connection helped them to manage psychological distress (Judge et al., 2001). In other words, identification with a team is a resource that can have positive outcomes on the health of staff (Kang et al., 2018). In the present context, care unit identification may be defined as the extent to which nurses felt part of a working group with a specific purpose, as well as part of a wider professional community (Caricati et al., 2013, 2015, 2020).

Some researchers (Sangal et al., 2021) have suggested that the sharing of workloads and an awareness of group cohesion help to build a sense of belonging and protect against stress and burnout. The adoption of measures based on autonomy, competence, and relatedness have encouraged nurses to seek support from supervisors and co-workers (Tang et al., 2020). Several other studies (e.g., Barello et al., 2020) have shown how social support and empathy help to reduce stress-related symptoms. Based on these findings, the main aim of the present study was to analyze the impact of COVID-19 on hospital nurses and to identify protective resources that might prevent psychological distress and burnout. The role of perceptions of safety in increasing work engagement and reducing stress and the moderating effect of care unit identification were investigated. It was hoped that the findings could be used to identify resources for the implementation of emergency interventions and human resource plans.

## Materials and Methods

### Hypotheses

Based on the motivational process of JDR model explained above, we hypothesized that the perception of safety would have both a direct relationship with burnout (H1) and emotional psychological distress (H2) and an indirect effect through the mediation of work engagement (H3). This presupposed a relation between work engagement and two negative outcomes: burnout (H4) and psychological distress (H5). Particularly, according to Ahmed et al. (2020), we hypothesized that the relationship between safety perception and burnout was mediated by work engagement. Secondly, we hypothesized that work engagement has a mediational role between safety perception and psychological distress.

**Figure 1:**
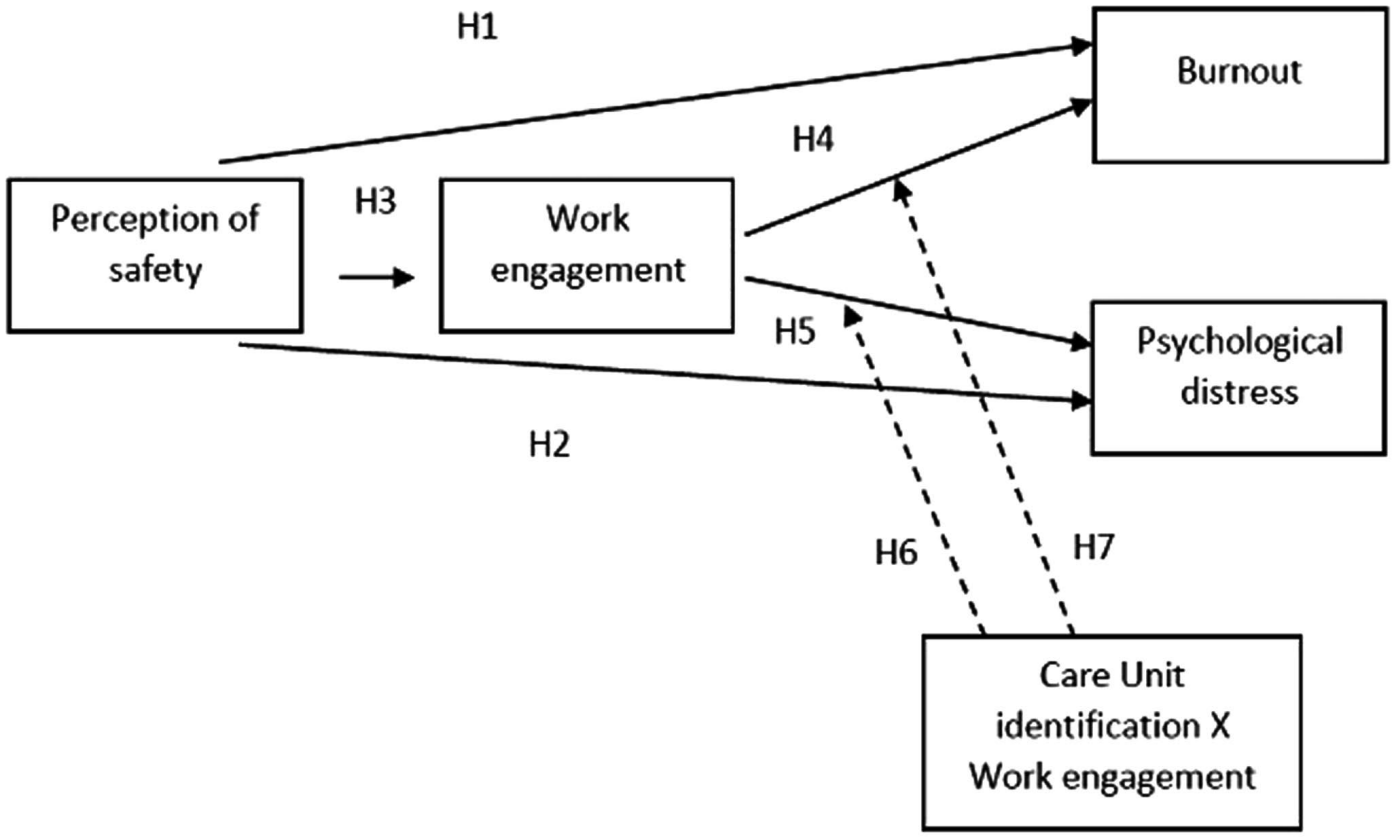
The hypothesized model. Solid lines represent supposed direct and mediational effects. Dashed lines represent the hypothetical moderation effects of CU identification on relationship of Work engagement and negative outcomes (Burnout and Psychological distress).

According to the literature concerning team support and identification, we also tested whether the two mediating relationships of the previous hypotheses were moderated by identification with the care unit. In particular, we hypothesized that work engagement reduced emotional psychological distress (H6) and burnout (H7) in nurses with higher levels of care unit identification. Figure 1 depicts the tested model and expected paths.

### Design and Setting

The study adopted a cross-sectional research design, and the data were collected using an online questionnaire. Before data collection, we shared instrument and research aims with the hospital general direction that authorized administration of the questionnaire. It was carried out in accordance with the American Psychological Association (APA) and National Association of Psychology ethical standards for the treatment of human subjects. Participants were informed that their participation was voluntary, that they could withdraw at any time, and that their data would be treated anonymously. They were also asked to read the informed consent form and agree to their involvement before completing the survey. The datasets generated during and analyzed during the current study are available from the corresponding author on reasonable request.

### Participants

The study involved 218 nurses at a hospital in northern Italy. Of these, 77.1% worked in a COVID-19 care unit. Most of the participants (81.7%) were women. Age-wise, 28.4% of the sample were between 41 and 50; 25.2% were between 51 and 60; 23.9% were between 18 and 30; 20.2% were between 31 and 40; and 2.3% were between 61 and 70. Nearly half (41.9%) of the participants had worked in the hospital for 21–35 years; 25.7% for 6–20 years; 6.9% for more than 35 years; and 32.1% for less than 1 or up to 5 years. Data were collected from July to September 2020, after the end of the first wave of the pandemic.

### Instrument

The instrument used was a self-report questionnaire containing the following scales: *Perception of Safety*. The Perception of Safety was measured using 11 items adapted from questionnaire developed by Akinboro et al. (2012). The Italian translation of the scale was adapted to perception of COVID-19 risk of contagion during pandemic in the hospital. Sample items were “The operators protection form COVID-19 infection has a high priority for the company management” and “The hospital staff received an adequate training to protect themselves from COVID-19 contagion.” The perception of Safety was measured on a six-point Likert scale (1 = strongly disagree, 6 = strongly agree). The internal consistency of this scale was *α* = 0.88.

*Care Unit (CU) Identification* was measured with the Italian five items of Caricati et al. (2015); sample items were “Being member of my CU is important to me” and “I am proud to belong to my CU” (*a* = 0.95). CU identification scale was measured on a six-point Likert-type scale (1 = strongly disagree, 6 = strongly agree). The internal consistency of this scale was *α* = 0.96.

*Work engagement* was measured using nine items from the Italian version (Balducci et al., 2010) of the Utrecht Work Engagement Scale—UWES (Schaufeli et al., 2002). Sample items are: “When I get up in the morning, I feel like going to work” and “I am enthusiastic about my job.” Participant was asked to response using a Likert scale (0 = Never, 1 = once a week or less, 2 = few times a month, 3 = once a week, 4 = few times a week, and 5 = every day). The internal consistency of this scale was *α* = 0.87.

*Psychological Distress* was measured with the Italian version (Bottesi et al., 2015) of the Depression Anxiety Stress Scales-21 (DASS-21; Lovibond and Lovibond, 1995), which consist of 21 items measuring self-reported levels of anxiety (*a* = 0.84), depression (*a* = 0.87), and stress (*a* = 0.88). Items ask participants to indicate the extent to which they experienced negative emotional states in the last 7 days on a six-point Likert scale (1 = never, 2 = rarely, 3 = sometimes, 4 = often, 5 = very often, and 6 = always). We considered to total score of the scale as the index of psychological distress; the internal consistency of the whole scale was *α* = 0.91.

*Burnout* is measured using 10 items of the Italian version of the Professional Quality of Life Scale; ProQol Version_5 (Stamm, 2009). Sample items are: “I feel trapped by my job as a helper” and “I feel overwhelmed because my work load to seems endless.” All items were scored on a six-point scale ranging from 1 = never to 6 = always. The internal consistency of this scale was *α* = 0.71.

### Analysis Plan

Zero-order correlations (Pearson’s *r*) were firstly investigated to assess association among variables. We preliminary checked assumptions of multivariate normality using Henze–Zirkler test (Henze and Zirkler, 1990). The model was then tested with structural equation modeling on manifest variables considering burnout and distress as dependent variables, safety perception, and work engagement as independent variables and CU identification as moderator independent variable (see Figure 1). Variables involved in interactions were centered at their grand mean before being entered in the regression matrix. All analyses were performed using R (R Core Team, 2021), and structural equation model was tested with Lavaan package (Rosseel, 2012). As we did not know all parameters of the models to detect sample size, we run Monte Carlo simulation to estimate the frequency of significant paths (i.e., their power) considering estimates as starting values of the population. About 1,000 Monte Carlo replications with *n* = 218 were performed. Finally, we will report indexes (e.g., chi-square, comparative fit index, and RMSEA) of model for descriptive purposes only and for sake of transparency. Note that, however, we were not interested in the adequacy of the model as our primary interests were on the estimated effect of considered paths.

## Results

### Preliminarily Analysis

Table 1 shows zero-order correlation and descriptive statistics regarding the measured variables. As indicated, the perception of safety was positively related with both work engagement and Care Unit identification, which was in turn positively and significantly correlated one to another. On the contrary, the perception of safety and CU identification were negatively related with burnout but were not correlated with psychological distress. Finally, the relationship between work engagement and the two negative outcomes (psychological distress and burnout) was negative. Zero-order correlations also indicated that considered measures were not related with professionals’ gender. Professionals with a longer work experience in hospitals appeared to report less psychological distress and perceive more safety and be more engaged. No significant correlation appeared between working experience and both burnout and CU identification.

**Table 1:** Descriptive statistics and zero-order correlation of measures.

	*M*	*SD*	2	3	4	5	6	7	8
1. Perception of safety	3.84	1.06	0.36^**^	−0.06	−0.27^**^	0.23^**^	−0.08	0.15^*^	0.11
2. Work engagement	3.91	0.93	-	−0.23^**^	−0.36^**^	0.50^**^	−0.04	0.20^**^	0.14^*^
3. Psychological distress	2.22	0.89		-	0.45^**^	−0.01	0.12	−0.15^*^	−0.16^*^
4. Burnout	2.59	0.61			-	−0.24^**^	0.04	−0.05	0.03
5. Care unit identification	4.96	1.22				-	−0.06	−0.01	−0.06
6. Gender (0 = men)	-	-					-	0.20	0.11
7. Tenure in hospital	-	-						-	0.89^**^
8. Age	-	-							-

*p < 0.05; **p < 0.01. *N* = 218.

Normality analysis revealed that data departed from multivariate normality (HZ = 1.829, *p* < 0.001), and then, we estimated model using maximum likelihood estimation with robust standard error which is robust to, and accommodate for, violation of the normality assumptions (Rosseel, 2012).

### Model Testing

**Figure 2:**
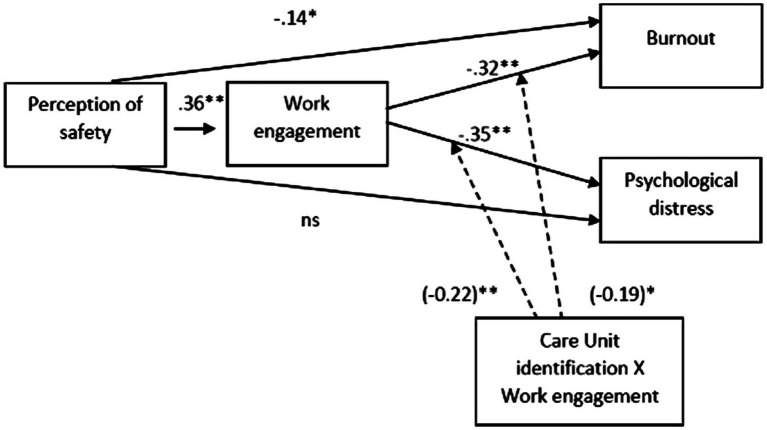
The tested model (standardized betas are reported). Solid lines represent direct and mediational effects. Dashed lines represent the moderation effects of CU identification on relationship of Work engagement and negative outcomes (Burnout and Psychological distress); interaction betas are reported in brackets.

General fit of the model appeared to be poor [*χ^2^*(2) = 51.13, *p* < 0.001, CFI = 0.725, RMSEA = 0.336, *p* < 0.001, 90%CI[0.269, 0.407]]. This is not surprising as we were not aiming to test a model which explained as much variance as possible. Results of the tested model are presented in Table 2. Perception of safety was positively and significantly related with work engagement, *b* = 0.315, *SE* = 0.060, *Z* = 5.252, *p* < 0.001, and negatively with burnout, *b* = −0.082, *SE* = 0.038, *Z* = −2.148, *p* = 0.032, while it had no significant relationship with psychological distress *b* = 0.028, *SE* = 0.058, *Z* = 0.490, *p* = 0.624. Work engagement showed a negative and significant relationship with both burnout, *b* = −0.209, *SE* = 0.051, *Z* = −4.097, *p* < 0.001, and psychological distress, *b* = −0.355, *SE* = 0.089, *Z* = −3.985, *p* < 0.001. CU identification, instead, had no significant relationship with burnout, *b* = −0.065, *SE* = 0.037, *Z* = −1.777, *p* = 0.076, nor psychological distress, *b* = 0.047, *SE* = 0.052, *Z* = 0.899, *p* = 0.369. Importantly, however, the CU identification interacted with work engagement in predicting both burnout, *b* = −0.077, *SE* = 0.034, *Z* = −2.284, *p* = 0.022, and psychological distress, *b* = −0.138, *SE* = 0.044, *Z* = −3.123, *p* = 0.002 (see Figure 2). When we unpacked these interactions, we discovered that the effect of work engagement on both burnout and psychological distress was stronger when CU identification was high (*b*_burnout_ = −0.303, *SE* = 0.074, *Z* = −4.082, *p* < 0.001; *b*_psychological distress_ = −0.523, *SE* = 0.127, *Z* = −4.126, *p* < 0.001) than when CU identification was low (*b*_burnout_ = −0.115, *SE* = 0.055, Z = −2.079, *p* = 0.038; *b*_psychological distress_ = −0.187, *SE* = 0.075, *Z* = −2.494, *p* = 0.013).

**Table 2:** Estimates from the structural equation modeling.

		*B*	*SE*	*Z*	95%CI	Beta	pwr
Work engagement							
	Safety perception	0.315	0.060	5.252^**^	[0.198; 0.433]	0.358	0.99
Burnout							
	Safety perception	−0.082	0.038	−2.148^*^	[−0.156; −0.007]	−0.142	0.57
	Work engagement	−0.209	0.051	−4.097^**^	[−0.309; −0.109]	−0.320	0.99
	CU identification	−0.065	0.037	−1.777	[−0.137; 0.007]	−0.131	0.49
	We × CU identification	−0.077	0.034	−2.284^*^	[−0.143; −0.011]	−0.186	0.77
Psychological distress							
	Safety perception	0.028	0.058	0.490	[−0.085; 0.142]	0.032	0.09
	Work engagement	−0.355	0.089	−3.985^**^	[−0.529; −0.18]	−0.355	1.00
	CU identification	0.047	0.052	0.899	[−0.055; 0.148]	0.061	0.16
	We × CU identification	−0.138	0.044	−3.123^**^	[−0.225; −0.051]	−0.218	0.86
							
Indirect effects							
	Security- > We- > Burnout	−0.066	0.020	−3.324^**^	[−0.105; −0.027]	−0.115	0.99
	Security- > We- > Distress	−0.112	0.035	−3.159^**^	[−0.181; −0.042]	−0.127	0.99

* *p* *< 0.05;*

** *p < 0.01*.

*We, Work engagement, CU, care unit, and pwr, power*.

*N = 218*.

Finally, we notice also that perception of safety had mediated indirect effects *via* work engagement on both burnout, *b* = −0.066, *SE* = 0.020, *Z* = −3.324, *p* = 0.001, and psychological distress, *b* = −0.112, *SE* = 0.035, *Z* = −3.159, *p* = 0.002, but these indirect effects were in turn moderated by CU identification as they were reduced for professionals who were weekly identified (*b*_burnout_ = −0.036, *SE* = 0.019, *Z* = −1.913, *p* = 0.056; *b*_psychological distress_ = −0.059, *SE* = 0.027, *Z* = −2.184, *p* = 0.029).

**Figure 3:**
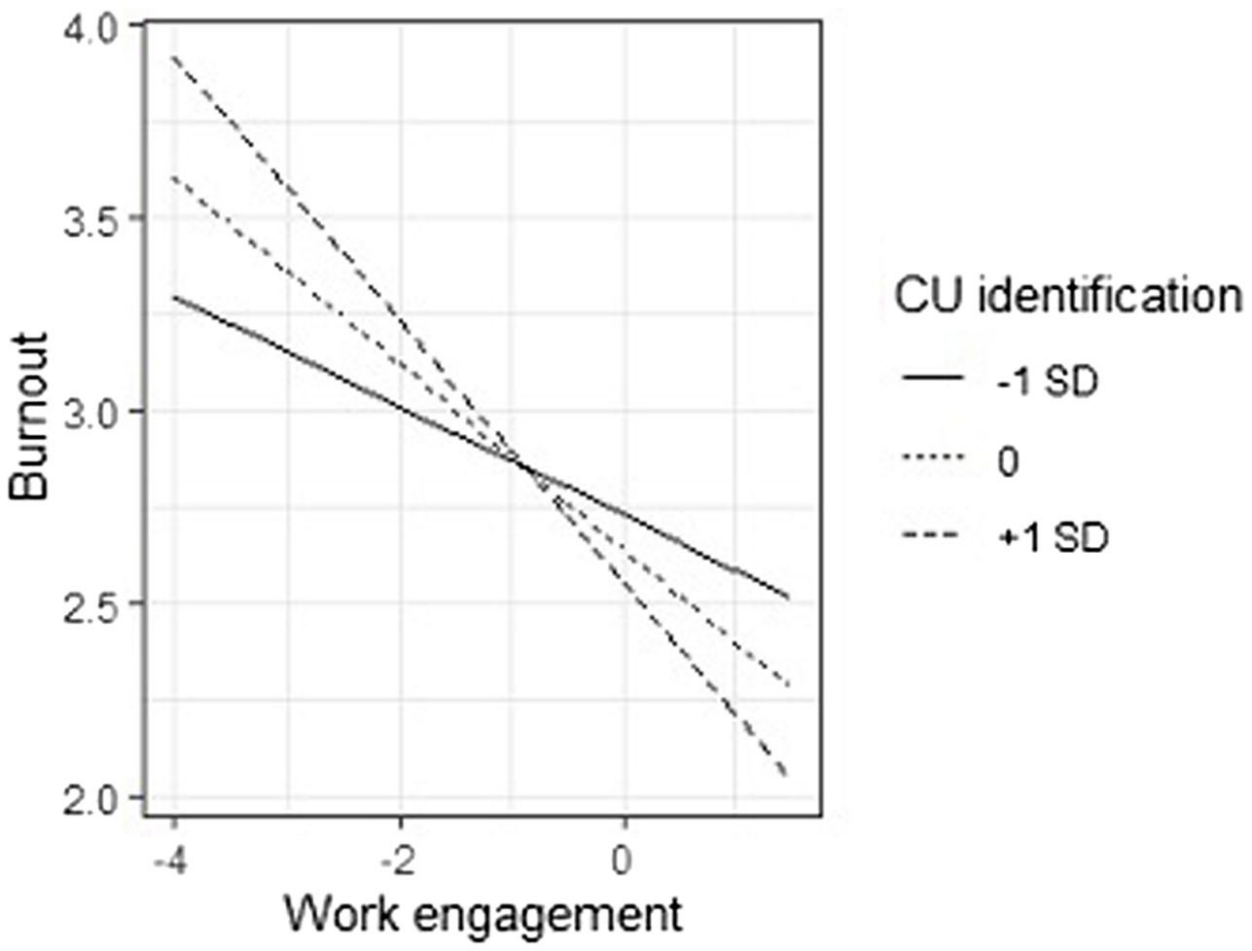
Interactions between Care Unit (CU) identification and Work engagement on Burnout.

**Figure 4:**
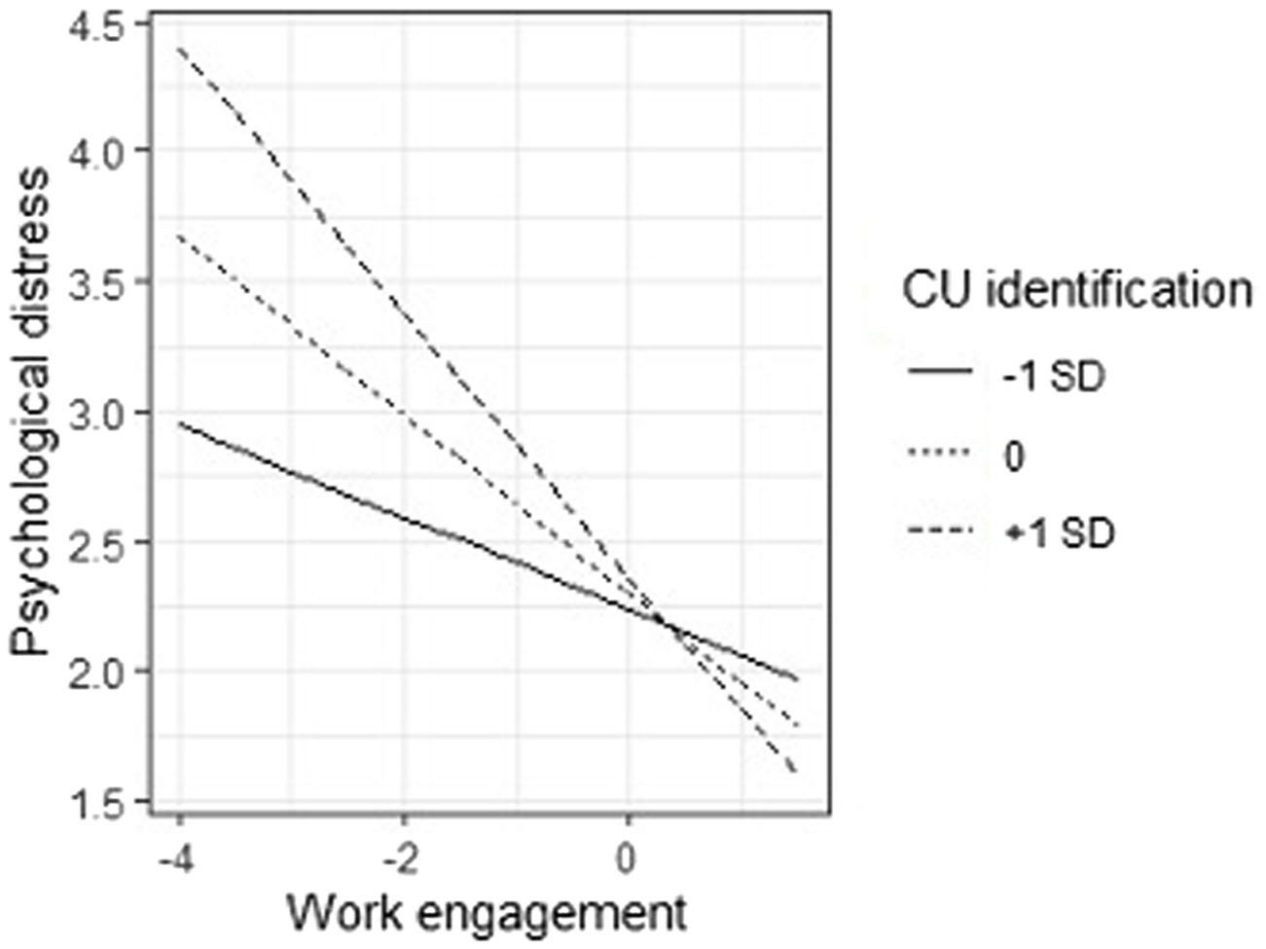
Interactions between CU identification and Work engagement on Psychological distress.

This model explained 12.8% of work engagement variance, 18.9% of burnout, and 17.6% of psychological distress (Figures 3, 4).

## Discussion

The pandemic has been a source of great stress both for individuals and groups. Health professionals represent one of the most affected categories. The present study aimed to investigate the consequences of the crisis on their wellbeing (nurses in particular) and to identify the protective resources that might prevent chronic psychophysical disorders.

Healthcare professionals often bear an excessive emotional burden due to the suffering of their patients, and they risk developing physical and psychological effects as a result of their strong emotional involvement. Factors, such as high workloads, difficult conditions, having to face stark choices, and the possibility of becoming ill personally (and seeing colleagues becoming ill) with no possibility of recovery, the great investment of energy, and the lack of personal space, are risk factors that can have a strong negative impact on health. However, the results of the present study show that the nurses did not experience too high levels of discomfort. Accordingly, the mean score of work engagement was high and, notably, in line with average scores that have been reported in studies carried out in both pandemic (e.g., Allande-Cussó et al., 2021) and pre-pandemic years (e.g., Van Bogaert et al., 2014; Ghazawy et al., 2019).

The present study has examined not only negative psychological outcomes but also the motivational processes and protective resources that have been neglected by the pandemic literature. In line with the JDR model, the results show that perceptions of security represented an important protective resource affecting the possibility of reacting by professionals and triggering a motivational process. In fact, this resource influenced their engagement, which in turn, forestalled the onset of burnout and psychological distress. The results support the hypothesis that work engagement played a mediating role between the perception of safety and stress.

A sense that the organization had invested in building a safe working environment helped staff react proactively to the challenges of the emergency, which diminished the risk of burnout and negative psychological symptoms (Vogus et al., 2020).

Another important protective resource was identification with the care unit. The present study shows how the working climate and team identification improved motivation in a time of crisis. A strong sense of belonging played a moderating role by strengthening the relationship between work engagement and stress symptoms and burnout. A high degree of identification with the work team encouraged collaboration and predicted positive health outcomes (Caricati et al., 2020). Furthermore, group identification and support enabled members to be aware of their emotions, share their perspectives, and be more efficient and focused (Barello and Graffigna, 2020).

### Limitations

The present study has several limitations. First, the use of cross-sectional questionnaires and a correlational design means that we must be cautious about inferring causal relationships between the variables. Second, the sample size was limited and consisted mostly of nurses, so the results cannot necessarily be generalized to other contexts.

## Conclusion

Most research on the pandemic has highlighted the risk factors and negative effects. The protective resources that might prevent symptoms of psychological distress have often been overlooked. The present study demonstrates that health professionals have shown high levels of vigor, dedication, and engagement in their work. Motivational factors have to be understood if psychological distress and burnout are to be prevented. The study has highlighted two resources (organizational and working group-orientated) that could be used in interventions. A safe and secure environment would help individuals manage adverse events by developing resilience and the skills needed to resolve underlying related issues, and an effort by organizations to encourage team identification initiatives would be similarly beneficial.

### Practical Implications

The results of the present study could help managers identify emergency planning resources. First, the study has management repercussions in terms of building effective teams at the micro-level. Managers should create a climate in which members feel safe, trustful of each other, and able to share knowledge and experiences. This will help teams work together to cope with the emergency. Second, from a governance perspective, the construction of a safe climate at a macro level will show workers that the organizational culture is supportive and attentive. Expectations of how adverse events should be interpreted and responded to can then be communicated more effectively.

## Data Availability Statement

The raw data supporting the conclusions of this article will be made available by the authors, without undue reservation.

## Ethics Statement

Ethical review and approval was not required for the study on human participants in accordance with the local legislation and institutional requirements. The patients/participants provided their written informed consent to participate in this study.

## Author Contributions

All authors contributed to the study’s conception and design. CP, LC, GG, NM, and AS performed material preparation and data collection. LC, CP, and CB performed analysis. CP, LC, and AB wrote the first draft of the manuscript. All authors contributed to the article and approved the submitted version.

## Conflict of Interest

The authors declare that the research was conducted in the absence of any commercial or financial relationships that could be construed as a potential conflict of interest.

## Publisher’s Note

All claims expressed in this article are solely those of the authors and do not necessarily represent those of their affiliated organizations, or those of the publisher, the editors and the reviewers. Any product that may be evaluated in this article, or claim that may be made by its manufacturer, is not guaranteed or endorsed by the publisher.
